# Bioactivities and genome insights of a thermotolerant antibiotics‐producing *Streptomyces* sp. TM32 reveal its potentials for novel drug discovery

**DOI:** 10.1002/mbo3.842

**Published:** 2019-04-02

**Authors:** Nareeluk Nakaew, Saisamorn Lumyong, William T. Sloan, Rungroch Sungthong

**Affiliations:** ^1^ Department of Microbiology and Parasitology, Faculty of Medical Science Naresuan University Phitsanulok 65000 Thailand; ^2^ Microbiology Division, Department of Biology, Faculty of Science Chiang Mai University Chiang Mai 50200 Thailand; ^3^ Infrastructure and Environment Research Division, School of Engineering University of Glasgow Glasgow G12 8LT UK

**Keywords:** antibiotics, antimicrobial resistance, bioactive compounds, genome mining, plant growth promotion, *Streptomyces*

## Abstract

A way to defeat antimicrobial resistance (AMR) crisis is to supply novel drugs to the pharmaceutical industry. This effort leads to a global call for seeking the beneficial microbes from underexplored habitats. To support this call, we isolated *Streptomyces* sp. TM32 from the rhizosphere soil of a medicinal plant, turmeric (*Curcuma longa* L.). TM32 exhibited strong antimicrobial activities against both human and plant pathogens, including an AMR pathogen, *Staphylococcus haemolyticus* MR‐CoNS. Surprisingly, such antimicrobial results of TM32's autoclaved crude extract remained the same. Based on the genome data analysis, TM32 belongs to the same genomic species with *Streptomyces sioyaensis* DSM 40032^T^, supported by the relatively high‐average nucleotide identity values (ANIb: 96.80% and OrthoANIu: 97.14%) and in silico DNA–DNA relatedness value of 75.40%. Importantly, the gene annotation analyses revealed that TM32's genome contains various genes encoding the biosynthesis of either known or unknown antibiotics and some metabolites involved in plant growth‐promoting traits. However, bioactivities and genome data comparison of TM32 and DSM 40032^T^ showed a set of apparent differences, for example, antimicrobial potentials, genome size, number, and occurrence of coding DNA sequences in the chromosomes. These findings suggest that TM32 is a new strain of *S*. *sioyaensis* and serves as an emerging source for further discovery of valuable and novel bioactive compounds.

## Graphical abstract

Streptomyces sp. TM32 can produce thermotolerant antibiotics that inhibit growth of bacteria and fungi. There are many gene clusters in the genome of TM32, encoding unknown secondary metabolites and plant growth‐promoting traits. TM32 is a potential source for novel drug discovery.

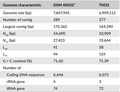

## 1 INTRODUCTION

The inappropriate use of antibiotics in medication and livestock, the anthropogenic release of natural and chemical biocides in agriculture, and the discharge of wastewater effluent containing bioactive residues to the environment, these altogether demonstrate the overuse of antimicrobial substances, which is a significant factor causing the antimicrobial resistance (AMR) crisis (Fiorentino et al., 2019; Rodriguez‐Mozaz et al., 2015; Ventola, 2015). Moreover, existing medicines are less efficient in the treatment of infectious diseases and have limitation for further developments of their modes of action, while the antimicrobial evolution of pathogens occurs more rapidly (Piddock, 2012; Spellberg, Bartlett, & Gilbert, 2013). These critical concerns lead to a global call for encouraging the discovery of novel drugs that possess distinct molecular structures and offer broad‐spectrum functions, as a chance to defeat the AMR problem.

The best‐known natural sources for seeking bioactive compounds (eg, antibiotics, anticancer/antitumor agents, biocatalysts, etc.) are plants and microbes. For centuries, some herbs have served as natural medicines in the treatments of human diseases (Alvin, Miller, & Neilan, 2014; Strobel & Daisy, 2003). These plants synthesize pharmaceutical metabolites that may be stored into their cells and tissues or released in the form of root exudates. Some microbes can live within or in contact with bioactive phytochemicals (Alvin et al., 2014; Nakaew & Sungthong, 2018; Strobel & Daisy, 2003), while some of which also enable to form the same or similar metabolites to those produced by plants (Alvin et al., 2014; Strobel & Daisy, 2003). These plant‐associated niches are yet a challenging source for the discovery of new microbial candidates and their novel secondary metabolites.

With the industrial point of view, antibiotic‐producing microbes require less space for cultivation, shorter time for biosynthesis, and offer higher flexibility in further biotechnological developments. Among the microbial producers, actinobacteria are well‐known cell factories for drug discovery, especially for those belonging to the genus *Streptomyces* (Bentley et al., 2002; Tiwari & Gupta, 2012). More than two‐thirds of all existing natural antibiotics derive from this actinobacterial genus (Bentley et al., 2002). *Streptomyces* is a well‐explored genus, which consists of 848 species and 38 subspecies, referring to the list of prokaryotic names with standing in nomenclature (http://www.bacterio.net/streptomyces.html) during the time of writing this article. Regarding these high numbers of published *Streptomyces* members, the recent trend of drug discovery is more focusing on other microbial resources, such as non‐*Streptomyces* actinobacteria, so‐called “rare actinomycetes” with the aim to avoid the repetitive finding of the formerly found bioactive metabolites (Tiwari & Gupta, 2012). However, hitherto, it is just a minority of microbes discovered, while the majority of them is hidden in nature and waiting for exploration.

*Streptomyces* sp. TM32 was isolated based on the concept of plant–microbe interactions from the rhizosphere soil of turmeric (*Curcuma longa* L.), a rhizomatous and herbaceous plant, often used in cooking (Nakaew, Rangjaroen, & Sungthong, 2015). A preliminary identification of TM32 using its 16S rRNA gene sequence revealed the closest phylogenetic relation to *Streptomyces sioyaensis* DSM 40032^T^, a producer of a peptide thiazole antibiotic, siomycin A, possessing both antibacterial and anticancer functions (Gartel, 2013; Tori et al., 1979). TM32 showed strong antifungal activity and was proven for its plant growth‐promoting (PGP) potentials in the suppression of phytopathogenic fungus, *Rigidoporus* sp. (Nakaew et al., 2015).

In this study, we aim to report some interesting bioactivities and the draft genomes of the type strain, *S*. *sioyaensis* DSM 40032^T^, and our emerging strain, TM32. We also unveil the biotechnological treasures in both genomes by employing some bioinformatics tools and discuss the novelty of TM32 based on a set of comparative genome data.

## 2 MATERIALS AND METHODS

The antimicrobial activities of crude extracts derived from DSM 40032^T^ (type strain) or TM32 were tested against either bacteria or fungi and some of which are pathogens of human and plant (Table 1). Each *Streptomyces* strain was grown in Hickey‐Tresner agar at 30°C for 7 days, which was transferred to grow further in 200 ml of International *Streptomyces* Project medium II (ISP2) broth under shaking conditions at 150 rpm, 30°C for 14 days (Nakaew et al., 2015). The whole liquid culture was extracted with ethyl acetate at a solute and solvent ratio of 1:2 (v/v) for one night, in which the separated supernatant was collected and evaporated at 45°C using a rotary evaporator (Büchi, Switzerland). The crude extracts obtained were divided into two proportions and one of which was heated by autoclaving at 121°C, 15 psi for 15 min.

**Table 1:** Some antimicrobial activities of crude extracts derived from *Streptomyces sioyaensis* DSM 40032^T^ and *Streptomyces* sp. TM32

Test microorganism	DSM 40032^T^ a		TM32^a^	
	Crude extract	Heated‐crude extract	Crude extract	Heated‐crude extract
Gram‐positive bacteria				
*Bacillus subtilis* DMST 5871	+ (8.0 ± 0.0)	+ (7.5 ± 0.7)	+ (8.0 ± 0.0)	+ (8.5 ± 0.7)
*Staphylococcus aureus* TISTR 1466	+ (9.5 ± 0.7)	+ (10.0 ± 0.0)	+ (9.5 ± 0.7)	+ (10.0 ± 0.0)
*Staphylococcus haemolyticus* MR‐CoNS 102	−	−	+ (8.0 ± 0.0)	+ (8.0 ± 0.0)
Gram‐negative bacteria				
*Escherichia coli* TISTR 887	−	−	−	−
Phytopathogenic fungi				
*Alternaria alternata* TISTR 3435	+ (6.0 ± 0.0)	+ (2.0 ± 0.0)	++++ (37.5 ± 0.7)	+++ (29.0 ± 1.4)

a The antimicrobial activity was determined into five levels (excellent ++++, very good +++, good ++, fair +, and no activity −). The value in parenthesis refers to the average size (ø mm) ± standard deviation of the inhibitory zones derived from the duplicate antagonism assays.

Both nonheated and heated crude extracts were dissolved with dimethyl sulfoxide (DMSO) and tested for their antimicrobial activities by the paper disk‐diffusion method described elsewhere (Nakaew, Sungthong, Ortega‐Calvo, & Lumyong, 2012). The overnight culture of test bacteria in nutrient broth (Himedia, India) and 2‐day‐old culture of test fungi in potato dextrose broth (Himedia, India) were prepared by shaking incubation at 150 rpm and 30°C. The culture of each test microbe was swabbed over its corresponding agar medium mentioned before. Paper disks were pasted on the test agar medium, in which 30 μL of the crude extracts, 2 mg/ml nalidixic acid (positive antibacterial), 1 mg/ml cycloheximide (positive antifungal), or DMSO (negative control) was dropped. The antimicrobial activity was reported with the diameter size of the inhibitory zone appeared on the assayed plates.

The biomass of DSM 40032^T^ or TM32 was prepared after cultivation in ISP2 broth under shaking conditions at 150 rpm, 30°C for 5 days. Total genomic DNA of both strains was extracted and used for sequencing with the Illumina HiSeq2500 system, following the services provided by BaseClear BV (Leiden, the Netherlands). The reads generated from the sequencing were assembled using SPAdes version 3.9 (Bankevich et al., 2012) and evaluated by QUAST version 3.2 (Gurevich, Saveliev, Vyahhi, & Tesler, 2013). The gene annotation analysis was carried out based on all contigs of each genome using Prokka (Seemann, 2014). The secondary metabolite‐related genes and gene clusters present in the genomes were predicted using antiSMASH 3.0 (Weber et al., 2015).

Identification of TM32 using its 16S rRNA gene sequence (GenBank accession no. KR534214) was performed using EzBioCloud database (https://www.ezbiocloud.net). The average nucleotide identity between the two genomes by BlastN (ANIb) and the orthologous average nucleotide identity by USEARCH (OrthoANIu) were computed in JSpeciesWS (Richter, Rosselló‐Móra, Oliver Glöckner, & Peplies, 2015) and EzBioCloud (Yoon, Ha, Lim, Kwon, & Chun, 2017), respectively. DNA–DNA relatedness between the two genomes was evaluated by in silico DNA–DNA hybridization (DDH) (Meier‐Kolthoff, Auch, Klenk, & Göker, 2013).

## 3 RESULTS

The crude extracts provided by ethyl acetate extraction of both *Streptomyces* strains did not show antimicrobial activity against the test Gram‐negative bacterium, *Escherichia coli* TISTR 887 (Table 1). Surprisingly, their heated crude extracts by autoclaving at 121°C remained antimicrobial functions. DSM 40032^T^ exhibited less antifungal activity than TM32 and did not inhibit a methicillin‐resistant coagulase negative pathogen, *Staphylococcus haemolyticus* MR‐CoNS 102 (Seng et al., 2017). The antifungal potential of DSM 40032^T^'s crude extracts was decreased by 67% when the extracts got heated before test, which was much higher compared to that of TM32 (23%). These findings highly prove that TM32 would serve as a promising source for further elucidations of valuable and novel antibiotics.

The sequencing generated 10,958,683 reads for DSM 40032^T^ and 6,613,213 reads for TM32. The characteristics of both genomes are listed in Table 2. DSM 40032^T^ has a genome size of 7,847,945 bp in 289 contigs with a G + C content of 71.60%, which is larger than the genome of TM32 (6,909,112 bp in 277 contigs with a G + C content of 71.39%). There are 6,646 coding DNA sequences (CDSs) predicted in the chromosome of DSM 40032^T^, including 74 tRNA and 4 rRNA genes, while TM32's chromosome contains 6,072 CDSs with 72 tRNA and 3 rRNA genes (Table 2).

**Table 2:** Genome characteristics of *Streptomyces sioyaensis* DSM 40032^T^ and *Streptomyces* sp. TM32

Genome characteristic	DSM 40032^T^	TM32
Genome size (bp)	7,847,945	6,909,112
Number of contig	289	277
Largest contig (bp)	175,362	144,590
*N*_50_ (bp)	54,690	33,909
*N*_75_ (bp)	27,423	19,644
*L*_50_	41	58
*L*_75_	94	124
G + C content (%)	71.60	71.39
Number of		
Coding DNA sequence	6,646	6,072
rRNA gene	4	3
tRNA gene	74	72

Some CDSs of both genomes have relevance in PGP traits, that is, nitrogen assimilation, phosphate solubilization, iron sequestration, and productions of biocatalysts and phytohormones (Table 3). TM32 possesses an extra chitinase gene (*chiD*), which is absent in the chromosome of DSM 40032^T^. This finding may result in the stronger antifungal activity of TM32 compared to DSM 40032^T^ (Table 1). However, TM32 lacks the exo‐β‐1,3‐glucanase gene, which is present in DSM 40032^T^'s chromosome. The absence of the glucanase gene was concordant to the phenotype of TM32 previously observed by an in vitro enzymatic assay (Nakaew et al., 2015).

**Table 3:** Some plant growth‐promoting gene clusters in the chromosomes of *Streptomyces sioyaensis* DSM 40032^T^ and *Streptomyces* sp. TM32

Plant growth‐promoting trait	Relevant gene and product		COG^a^	DSM 40032^T^ b	TM32^b^
Nitrogen assimilation	*nasD*	Nitrite reductase	COG1251	+	+
	*napA*‐1	Nitrate reductase		+	+
	*napA*‐2	Nitrate reductase		+	−
	*narK*‐1	Nitrate/nitrite transporter	COG2223	+	+
	*narK*‐2	Nitrate/nitrite transporter	COG2223	+	−
Phosphate solubilization	*gpm2*‐1	Acid phosphatase	COG0406	+	+
	*gpm2*‐2	Acid phosphatase	COG0406	+	+
	*phoD*‐1	Alkaline phosphatase D	COG3540	+	+
	*phoD*‐2	Alkaline phosphatase D	COG3540	+	−
	*phoP*‐1	Alkaline phosphatase synthesis transcriptional regulatory protein	COG0745	+	+
	*phoP*‐2	Alkaline phosphatase synthesis transcriptional regulatory protein	COG0745	+	+
	*phoP*‐3	Alkaline phosphatase synthesis transcriptional regulatory protein	COG0745	+	−
	*sphR*	Alkaline phosphatase synthesis transcriptional regulatory protein	COG0745	−	+
	*ycdX*	Phosphatase		+	+
		Putative phosphatase		+	+
		Putative phosphatase	COG0560	+	−
Iron sequestration	*sbnA*	Putative siderophore biosynthesis protein	COG0031	+	+
	*yusV*	Putative siderophore transport system ATP‐binding protein	COG1120	+	+
	*yfhA*	Putative siderophore transport system permease protein	COG0609	+	+
	*yfiY*	Putative siderophore‐binding lipoprotein	COG0614	+	+
Biocatalyst	*acdS*	1‐Aminocyclopropane‐1‐carboxylate deaminase		+	+
	*aml*	α‐Amylase		+	+
	*bca*‐1	Bromoperoxidase‐catalase		+	+
	*bca*‐2	Bromoperoxidase‐catalase		+	+
	*katA*	Catalase		+	+
	*katG*	Catalase‐peroxidase		+	+
	*cenA*	Endoglucanase A (endo‐β‐1,4‐glucanase)	COG5297	+	+
		Exo‐β‐1,3‐glucanase		+	−
		Exochitinase 1		+	−
	*chiA*‐1	Putative bifunctional chitinase/lysozyme		+	+
	*chiA*‐2	Putative bifunctional chitinase/lysozyme		−	+
	*chiC*	Chitinase C		+	+
	*chiD*	Chitinase D		−	+
		Lipase		+	+
		Lipase 2		+	+
	*lip3*‐1	Lipase 3		+	+
	*lip3*‐2	Lipase 3		+	+
		Thermostable monoacylglycerol lipase		+	+
	*snpA*	Extracellular small neutral protease		+	+
	*htpX*‐1	Protease	COG0501	+	+
	*htpX*‐2	Protease		+	+
	*htpX*‐3	Protease	COG0501	+	+
	*htpX*‐4	Protease		+	+
	*htpX*‐5	Protease		+	−
	*prtS*	Protease		+	+
Phytohormone production	*trpC*	Indole‐3‐glycerol phosphate synthase	COG0134	+	+

a COG refers to “Clusters of Orthologous Groups” in the Prokka pipeline (Seemann, 2014).

b The presence and absence of each gene cluster is determined by + and −, respectively.

DSM 40032^T^ has a higher number (45) of secondary metabolite‐related gene clusters than TM32 (29 gene clusters) (Table 4). Both strains have a siomycin‐encoding gene cluster in their genomes, which is a unique characteristic of *S*. *sioyaensis* (Gartel, 2013; Tori et al., 1979). DSM 40032^T^ possesses a lot of micromonolactam gene clusters (8), while TM32 does not have even one. The PGP‐related siderophore gene clusters are present in both genomes. Interestingly, there are many gene clusters of unknown secondary metabolites found in both genomes (indicated with <100% gene cluster similarity to the database), which should be elucidated later for their bioactivities, molecular structures, and biosynthetic pathways.

**Table 4:** Some gene clusters encoding secondary metabolite biosynthesis in the chromosomes of *Streptomyces sioyaensis* DSM 40032^T^ and *Streptomyces* sp. TM32

Type of secondary metabolite	Most similar known cluster	MIBiG BGC‐ID^a^	DSM 40032^T^ b	TM32^b^
Bacteriocin			+ (3 clusters)	+ (3 clusters)
Butyrolactone	Blasticidin	BGC0000874	+ (7%)	+ (7%)
	Coelimycin	BGC0000038	+ (12%)	−
	Unknown		+ (1 cluster)	+ (1 cluster)
Butyrolactone‐Phenazine‐other PKS	Esmeraldin	BGC0000935	+ (64%)	−
Ectoine	Ectoine	BGC0000853	+ (100%)	+ (100%)
Ladderane	Colabomycin	BGC0000213	+ (4%)	+ (4%)
Lanthipeptide	Kanamycin	BGC0000703	−	+ (1%)
	Unknown		−	+ (1 cluster)
Lassopeptide	Chaxapeptin	BGC0001307	+ (42%)	+ (42%)
	FD‐594	BGC0000222	−	+ (4%)
Linaridin	Legonaridin	BGC0001188	+ (55%)	+ (55%)
	Unknown		+ (1 cluster)	−
Melanin	A‐500359s	BGC0000949	+ (5%)	−
Non‐ribosomal peptide synthase (NRPS)	A‐503083	BGC0000288	+ (7%)	−
	Lipstatin	BGC0000382	+ (28%)	+ (28%)
	Neocarzilin	BGC0000111	−	+ (14%)
	Unknown		−	+ (1 cluster)
Polyketide synthase (PKS)				
Type 1 PKS	Hedamycin	BGC0000233	+ (6%)	−
	Maklamicin	BGC0001288	+ (13%)	−
	Micromonolactam	BGC0000095	+ (100%, 8 clusters)	−
	Phoslactomycin B	BGC0000123	+ (100%)	−
	Pladienolide	BGC0000126	+ (50%)	−
	PM100117/PM100118	BGC0001359	+ (26%, 40%, 47%)	−
	Stambomycin		+ (36%)	−
	Unknown	BGC0000151	+ (1 cluster)	+ (1 cluster)
	Vicenistatin	BGC0000167	+ (25%, 60%)	−
Type 2 PKS	Lysolipin	BGC0000242	−	+ (50%)
	Spore pigment	BGC0000271	+ (75%)	+ (75%)
Type 3 PKS	Naringenin	BGC0001310	+ (100%)	+ (100%)
Betalactone‐NRPS‐Type 1 PKS	Lomaiviticin	BGC0000240	−	+ (3%)
Other PKS‐Type 1 PKS	Roseoflavin	BGC0000927	+ (100%)	−
	Sanglifehrin A	BGC0001042	−	+ (4%)
	Unknown		+ (1 cluster)	+ (1 cluster)
Trans‐AT PKS	Dorrigocin/Migrastatin	BGC0000177	+ (100%)	−
Siderophore	Desferrioxamine B	BGC0000941	+ (80%)	+ (80%)
	Unknown		+ (2 clusters)	+ (2 clusters)
Terpene	Hopene	BGC0000663	+ (61%)	+ (69%)
	Isorenieratene	BGC0000664	+ (71%)	−
	Salinomycin	BGC0000144	−	+ (6%)
	Unknown		−	+ (1 cluster)
Thiopeptide‐Terpene	Siomycin	BGC0000611	+ (100%)	+ (100%)
Total			45 clusters	29 clusters

a MIBiG refers to “Minimum Information about a Biosynthetic Gene cluster” in the antiSMASH 3.0 (Weber et al., 2015).

b The presence (+) or absence (−) of each gene cluster is determined, while the value indicated in the parenthesis is the similarity percentage to the known cluster or the number of cluster found.

Although the 16S rRNA gene sequence similarity of TM32 and DSM 40032^T^ was 99.23%, this percentage is insufficient to determine the difference in genomic species. Therefore, the average nucleotide identity values (ANIb and OrthoANIu) between the genomes of TM32 and DSM 40032^T^ were computed, whereas ANIb and OrthoANIu were 96.80% and 97.14%, respectively. Besides, DNA–DNA relatedness value between the two genomes evaluated by in silico DDH was 75.40%. Following the recent standards for using the genome data in the taxonomy of prokaryotes proposed by Chun et al. (2018), TM32 belongs to the same species with DSM 40032^T^. However, the apparent differences in bioactivities and genotypes of both strains would be sufficient to propose TM32 as a new subspecies of *S*. *sioyaensis*, in which a set of polyphasic approaches should be performed further to complete the taxonomy of TM32.

We believe that our proposing new strain of *Streptomyces* TM32 with its potentials to form thermotolerant antibiotics and genome insights could be a challenging cell factory that advances various biotechnological applications, for instance, novel drug discovery to defeat AMR crisis and green technology to optimize organic agriculture.

## CONFLICT OF INTERESTS

The authors declare not to have any conflict of interest.

## AUTHOR CONTRIBUTIONS

NN provided the bacteria, performed bioactivity tests, and prepared the manuscript. SL and WTS gave advices regarding the work. RS supervised the work, wrote and edited the manuscript.

## ETHICS STATEMENT

None required.

## Data Availability

These whole genome shotgun projects have been deposited at DDBJ/ENA/GenBank under the accessions SDIF00000000 (BioProject: PRJNA516206) and SDIG00000000 (BioProject: PRJNA516236) for *Streptomyces sioyaensis* DSM 40032^T^ and *Streptomyces* sp. TM32, respectively. The versions described in this paper are SDIF01000000 and SDIG01000000, accordingly.
